# Compact Design and Image-Space Pose Control of a Robot for Tendon-Driven Concentric Catheters in Mitral Repair Interventions

**DOI:** 10.1109/TMECH.2025.3602061

**Published:** 2026-04

**Authors:** Weizhao Wang, Zhouyang Xu, Aya Mutaz Zeidan, Carlo Saija, Yixuan Zheng, Matteo Arena, Shuangyi Wang, Richard James Housden, Kawal Rhode

**Affiliations:** School of Biomedical Engineering and Imaging Sciences, King’s College London, WC2R 2LS London, U.K.; State Key Laboratory of Multimodal Artificial Intelligence Systems, Institute of Automation, Chinese Academy of Sciences, Beijing 100190, China; School of Artificial Intelligence, University of Chinese Academy of Sciences, Beijing 101408, China; Centre for Artificial Intelligence and Robotics, Hong Kong Institute of Science & Innovation, Chinese Academy of Sciences, Hong Kong; School of Biomedical Engineering and Imaging Sciences, King’s College London, WC2R 2LS London, U.K.

**Keywords:** Tendon-driven concentric catheter, image space, pose control, closed-loop control, mitral edge-to-edge repair

## Abstract

Accurate clip positioning (including position, direction, and orientation) in transcatheter mitral edge-to-edge repair (M-TEER) is challenging due to the complexity of manipulating tendon-driven concentric catheters under imaging guidance. Existing methods are limited to either task-space position/shape control or image-space joint control. This study presents a compact eight-degree-of-freedom robot for controlling the PASCAL device, achieving closed-loop pose control in image space. Building on a prior variable curvature model, we develop a kinematic model that accounts for coupling between catheter handles and compensates for tip length variation. A prior sheath shape model is also integrated to support safer navigation in confined anatomy during clip adjustment. To achieve precise image-space pose control, we design a novel framework featuring a mapping strategy for independent control of clip position, direction, and orientation relative to registered imaging views, along with a Jacobian-based PID controller. Simulation results demonstrate effective independent control, with only direction adjustments exhibiting minor coupling with orientation (maximum deviation: 2.69°). Experiments show that closed-loop control effectively mitigates catheter misalignment and rubber deformation, achieving average positional and angular RMSEs to 0.52 mm and 0.61°. This robot provides precise, image-space pose control, which is expected to assist with the challenge of accurate clip positioning in M-TEER.

## I Introduction

**M**ITRAL valve (MV) disease is one of the most prevalent valvular heart defects worldwide. Most patients present with mitral regurgitation (MR), a condition in which the MV fails to close properly, and its prevalence increases with age [1]. The mortality rate of untreated severe MR is up to 50% [2]. However, approximately 50% of patients who meet the indication are not referred to surgery due to factors such as heart failure, older age, or other comorbidities [3]. To address MR in patients at high surgical risk, catheter-based interventions have been developed over the past decade. In particular, transcatheter mitral edge-to-edge repair (M-TEER) has evolved from an experimental technique to a guideline-recommended, safe, and effective treatment. It is now the most commonly performed structural intervention for both severe primary and secondary MR [4].

**Fig. 1:**
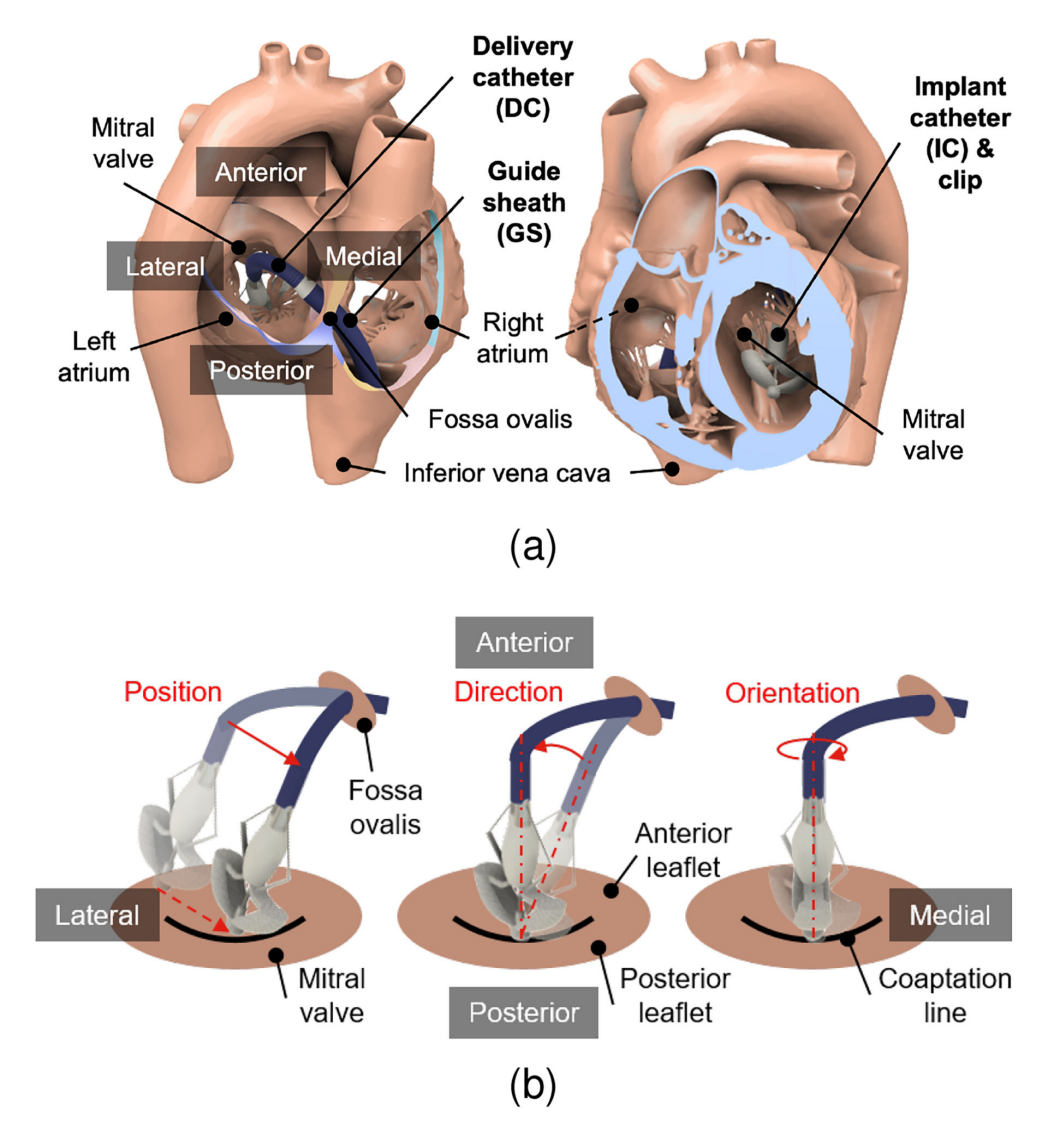
M-TEER demonstration (a) PASCAL navigation within relevant cardiac anatomy. (b) Adjustments of the clip’s position, direction, and orientation for optimal positioning.

Two delivery systems currently approved in Europe for M-TEER are MitraClip (Abbott Vascular, Santa Clara, CA, USA) and PASCAL (Edwards Lifesciences, Irvine, CA, USA) [4]. Both systems use a three-catheter design: a steerable guiding sheath (GS), a steerable delivery catheter (DC), and an implant catheter (IC) with a pre-attached clip at its tip (Fig. 1a). The GS is inserted via the femoral vein and advanced through the inferior vena cava, right atrium, across the fossa ovalis, and into the left atrium to establish the access route. The DC is then flexed toward the MV, and the IC delivers the clip to suture the prolapsing or flail leaflet to the opposing leaflet, creating a double or reduced orifice to mitigate MR [5], [6]. The GS and DC are controlled by tendons actuated via distal knobs that allow the bending. This setup represents a hybrid continuum structure with tendon-driven sections in a concentric assembly, providing extensive maneuverability within the left atrium [4]. Hereafter, it is referred to as ”tendon-driven concentric (TDC) catheters” as defined by Chikhaoui et al. [7].

M-TEER is guided by X-ray and transesophageal echocardiography (TEE), with certain TEE views used especially for clip positioning and leaflet grasping [8], [9]. For optimal positioning, the clip must be perpendicular to the coaptation line (where the leaflets meet during heart contraction) and aligned with the MV central region. Additionally, the DC and IC axes should be perpendicular to the anterior and posterior leaflets [10]. Technically, this requires 6 degrees of freedom (DoFs) and accurate adjustments in position, direction, and orientation (as defined in Fig. 1b). These adjustments are performed by advancing, retracting, rotating, or bending the TDC catheters, following the latest PASCAL manipulation guidelines [11]. However, the steerability of both the GS and DC is imperfect, and each adjustment often introduces secondary effects. For example, bending the DC moves the clip medially but also shifts its distal tip anteriorly. To compensate, the GS must be adjusted posteriorly, which in turn causes the system to move backward. These interdependent movements complicate the procedure and make precise clip positioning challenging [10]. Achieving independent control of the clip’s position, direction, and orientation relative to the imaging plane would greatly enhance the precision and efficiency of M-TEER and similar structural heart interventions.

In recent years, TDC robots have gained increasing attention as they combine the strengths of tendon-driven mechanisms and concentric tube designs. Table I summarizes representative studies from 2020 to 2025 on MV interventions and other purposes, in terms of modeling, DoFs, control targets, closed-loop controllers, use of image guidance, and applications. MV intervention robots can be categorized into (1) add-on systems integrated with commercial catheters and (2) complete custom platforms. Among the former, Zhang et al. [12] developed a 3-DoF system for MitraClip manipulation and achieved a mean position error of 1.29 mm using a Cosserat rod (CR) model and PID control. Due to the difficulty in estimating the Cosserat parameters, Bicchi et al. [13] used a simplified constant curvature (CC) model with a P controller, reaching a target position error of about 1.92 mm. However, neither method accounts for hysteresis, and accuracy could be further improved. SurgiPulse Robotics Co., Ltd. performed the first-in-human robot-assisted M-TEER using MitraClip in 2024 [16], although technical details remain undisclosed. For the latter category, Qi et al. [14] proposed a 4-DoF robotic transcatheter system for MV implant, achieving task space position and one orientation root mean square errors (RMSEs) of 1.00 mm and 2.00° with joint hysteresis compensation. Based on this, an ultrasound-guided joint-space control method was further proposed to enable real-time estimation of all joints [15]. Despite these advancements, current MV robotic systems are limited to 3–4 DoFs and provide restricted orientation control. Full clip pose control under image guidance has yet to be developed.

Beyond MV interventions, Jeong and Sarma [17], [18] developed a guidewire robot actuated by one tendon to control its arc length and curvature. However, the design cannot generate multiple independent bends, which are essential for navigating complex anatomies. Chitalia and Yamamoto [19], [20] introduced CR models for tendon-actuated concentric tubes with two bends. Wei and Zhang [21], [22] modeled the coupling effects between an inner concentric tube and an outer cable-driven continuum. However, they only presented the position control performance. To improve dexterity in confined spaces, Wang [23] proposed a robot for simultaneous control of an ablation sheath and catheter, and validated its pose control. Additionally, some researchers have proposed robots with 6 or more DoFs for whole-shape control, which also enables tip pose control [24], [25]. However, TEE or X-ray imaging offer limited field of view or only 2D information, making full-shape control difficult to implement. The main limitations of these systems when applied to M-TEER are summarized below.

1) **Modeling Limitations**: CR models are computationally intensive and rely on geometric and material parameters that are difficult to measure. CC models cannot accurately represent catheters with variable curvature.
2) **Tip Pose Control Limits**: Existing shape control methods can adjust the tip pose but are constrained in TEE or X-ray, where the imaging field is limited or 2D.
3) **Image-Guided Control at Joint Level**: Current image-guided robotic systems are limited to joint-space control. Precise and independent control of the clip pose in image space has not yet been achieved.

In response to these problems, this paper aims to develop a compact robot and control framework for image-space pose control of TDC catheters. We hypothesize that this approach enables independent and precise control of the clip’s position, direction, and orientation in image space. We first build upon a previous sheath robot [26] by redesigning it with a more compact structure and synchronized actuation to accommodate the PASCAL. We then build on our prior variable curvature model, which does not rely on material properties [27], to develop a kinematic model. This additionally accounts for the coupling between catheter handles and compensates for tip length variation. In addition, a sheath shape model [26] is further integrated to enhance safe sheath navigation through confined regions such as the fossa ovalis. Using this, we develop a framework for independent pose control in image space. The system is validated through simulations to demonstrate effective independent control followed by experiments to compare open- and closed-loop performance to assess positioning accuracy.

In this paper, the design, modeling, control, and validation of the robot are presented. These methods are detailed in Section II. Results are analyzed in Section III, while Section IV summarizes the conclusions and proposes future research directions. The contributions are summarized as follows:

1) A compact, 8-DoF robotic system for the PASCAL. It compensates for tip length variation and enables safe GS navigation through confined anatomical regions during clip adjustments.
2) An image-space pose control framework that combines i) a mapping strategy for independent control of clip position, direction, and orientation relative to imaging views, and ii) a Jacobian-based PID controller that ensures accurate closed-loop tracking.
3) Comprehensive validation through simulation and experiments, demonstrating effective independent control and significantly enhanced accuracy: closed-loop control reduced positional and angular RMSEs from 3.69 mm and 4.51° (open-loop) to 0.52 mm and 0.61°, respectively.

## II Methods

### A Compact Robotic System Design

**Fig. 2:**
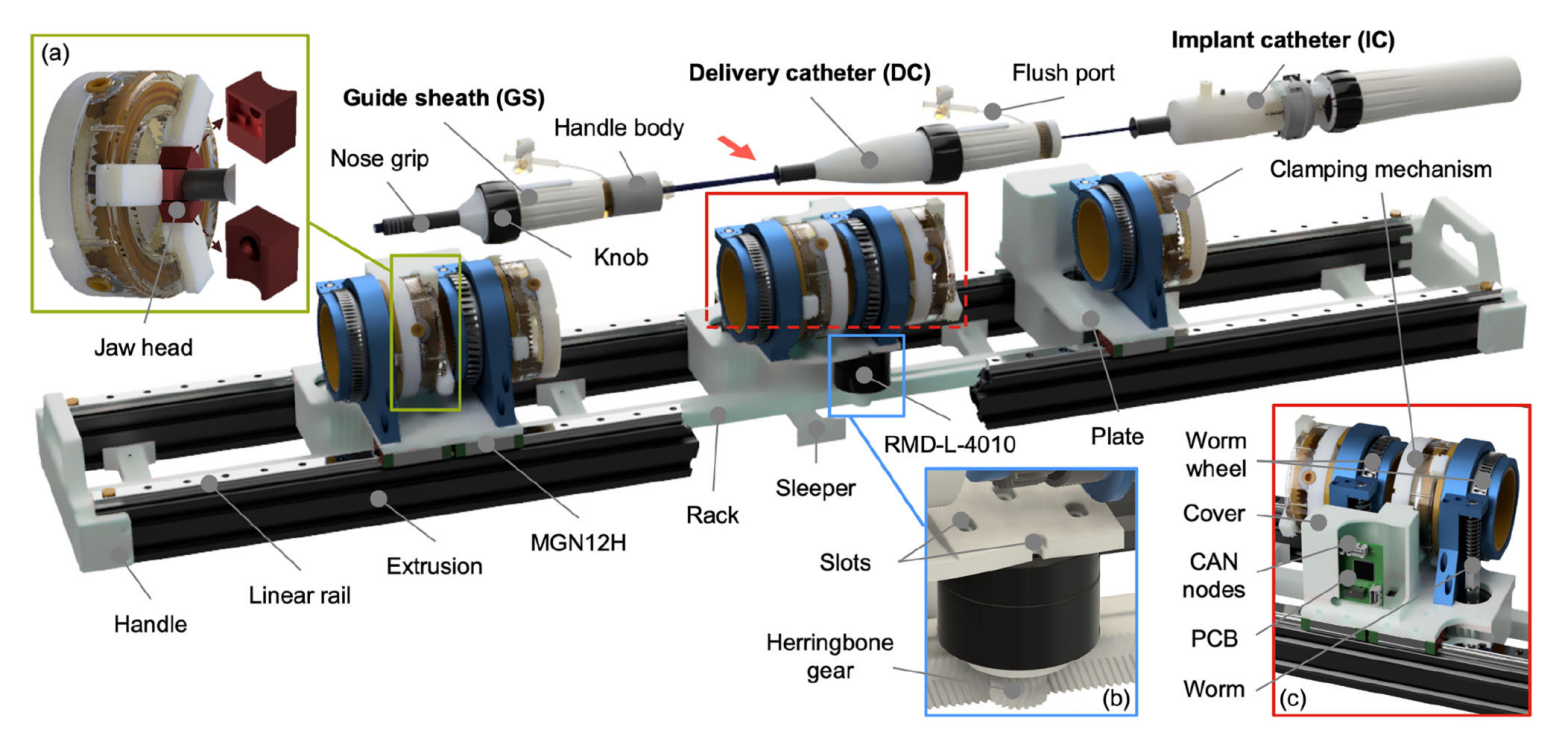
Robotic system for manipulating TDC catheters (PASCAL) (a) Clamping mechanism with customized jaw heads, each ensuring two secure contact points with the clamped object. (b) All motors mounted beneath the base plate to lower the center of gravity and streamline wiring. (c) Compact transmission system incorporating a worm gear mechanism with gear ratios of 1:77 for handle rotation and 1:46 for knob rotation, enabling precise joint synchronization.

Inspired by the stabilizer rail system (Edwards Lifesciences, Irvine, CA, USA) used for the PASCAL, the robotic system was enhanced from the previously developed GS robot [26] by adding two additional plates. This enabled simultaneous manipulation of the GS, DC, and IC (Fig. 2). In this clamping mechanism (Fig. 2a), the nose grip (rubber material) at the catheter front was clamped instead of the handle body. This was because: (1) the rubber’s higher friction prevents slippage during translational and rotational motions, and (2) this placement provides easier access to flush ports. To accommodate the different diameters of the nose grips and knobs, we designed a customized jaw head to create two contact points with the nose grip, ensuring an even distribution of gripping force. The estimated clamping force exceeds the axial and rotational force requirements in clinical use. If needed, gripping performance can be further improved by increasing applied torque or improving mechanical efficiency, such as through lubrication.

The translational mechanism was upgraded by replacing rail guide shafts with high-precision MGN12H linear rails mounted on two 20×40 mm^2^ extrusions. It has a transmission ratio of 40 mm per full motor rotation (Fig. 2b). Handles were added on both sides to facilitate manual transport. The belt transmission was replaced with a space-efficient worm gear mechanism (Fig. 2c). Given the larger turning range of the knobs compared to the handles, the transmission ratio for handle rotation was set to 1:77, while that for bending was set to 1:46. This configuration ensured joint synchronization. NEMA 17 motors were upgraded to smaller BLDC motors (RMD-L-4010, MyActuator Co., Ltd., Suzhou, China), all mounted under the base plate to lower the center of gravity. The motor’s nominal speed of 560 rpm ensures each joint’s speed meets clinical requirements. As a result, the robot’s overall dimensions were optimized: its width and height were reduced from 246×174 to 178×146 mm^2^ and its length increased from 985 to 1026 mm, enabling greater translational movement. This configuration provides the GS and DC with translation, rotation, and bending capabilities, and the IC with translation and rotation. It collectively offers a total of 8 DoFs, exceeding the 6 typically required for clinical use.

To streamline wiring for communication, a custom PCB was used to merge 2 or 3 Controller Area Network (CAN) bus [28] nodes on a single plate and output only one CAN bus pair cable (Fig. 2c). The system was controlled by a laptop running Ubuntu 20.04, which received and sent control commands. Among all components, the three motors responsible for translational movements had the highest current demand at approximately 1 A each. Considering the total system requirements, a 24 V, 5 A power supply (Joylit, Shenzhen Zhaolan Photoelectric Technology Ltd., Shenzhen, China) was chosen to ensure sufficient capacity.

In manufacturing, the primary focus was on preventing wear and minimizing backlash. All parts were 3D printed using PLA, except for the gear, rack, and worm, which were printed in Nylon to better withstand high forces. To address dimensional variability in 3D printing, a 0.2 mm tolerance was added to the worm gear components to balance slack reduction with long-term durability. Adjustable slots (Fig. 2b) were also incorporated to fine-tune the gear-rack distance, allowing for tension adjustments to minimize backlash.

### B Kinematic Modeling and Compensation

**Fig. 3:**
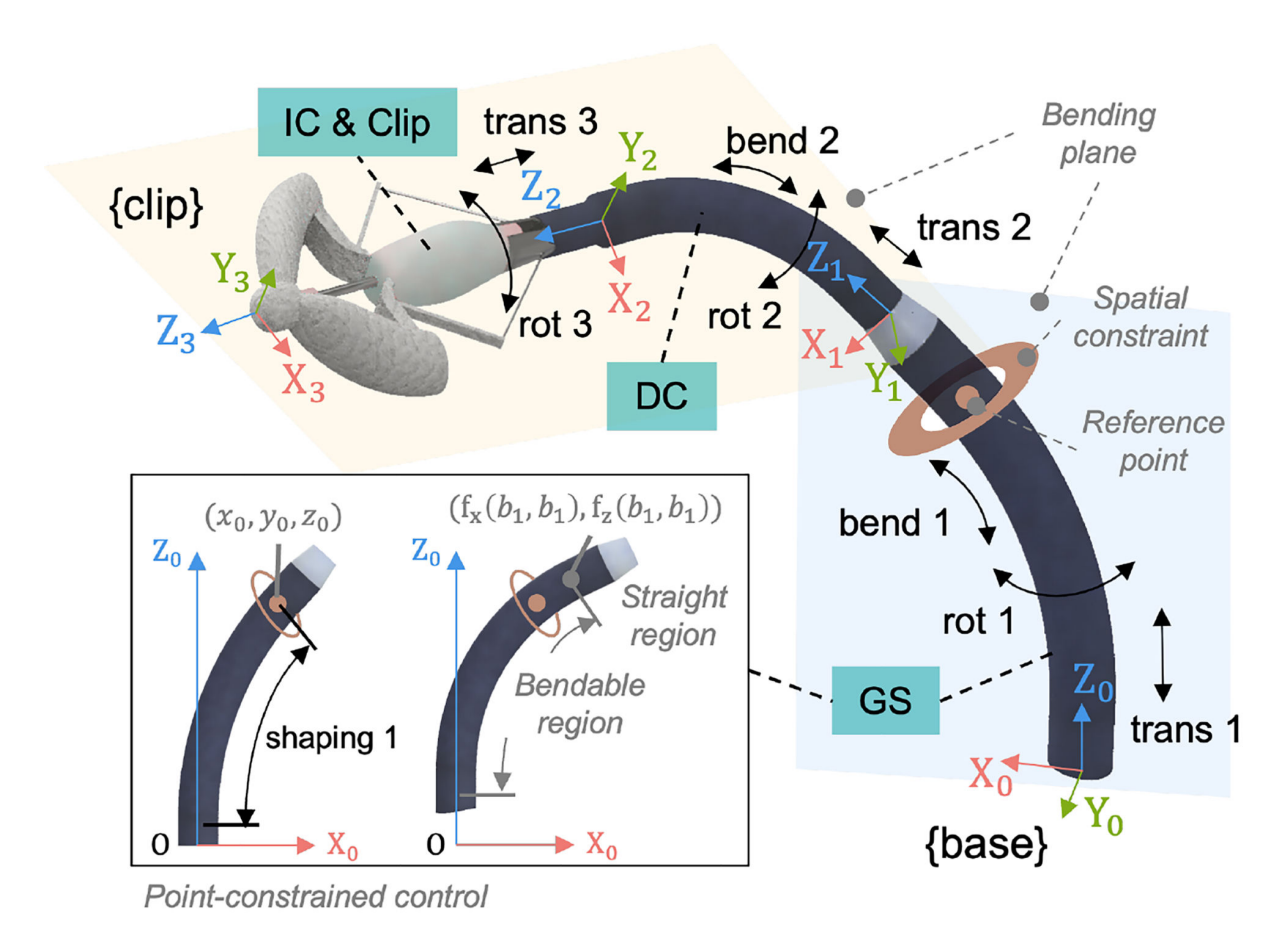
Clip model with defined coordinate systems and DoFs in the configuration space: The black box illustrates the pseudo-DoF to adjust the GS shape.

Based on tip deformation patterns, these catheters are modeled as a 9-DoF TDC robot (Fig. 3), containing one pseudo-DoF for GS shape calculation. Coordinate systems are defined with origins at the end of each tip section, where bending occurs in the X-O-Z plane and the Z-axis aligns with the catheter centerline. In the figure, coordinate system 0 represents the base, systems 1 and 2 represent the GS and DC coordinate frames, respectively, and system 3 corresponds to the IC and clip coordinate frame. The clip’s total movement is as follows: the GS and DC each have three DoFs (translation, rotation, and bending), and the IC has two DoFs (translation and rotation).

Additionally, a pseudo-DoF is introduced to determine the position of shape along the GS, called ”shaping”. This variable is expressed in radians within the bendable region to indicate curvature and in millimeters within the straight region to indicate length (see black box in Fig. 3). To support safe navigation through spatial constraints (e.g., the fossa ovalis) during clip positioning, a point-constrained control strategy [26] is incorporated. For clarity, this paper uses bold black letters for vectors and matrices, italic letters for variables, and Roman style for non-variable labels. This strategy defines a reference point (*x*_0_, *y*_0_, *z*_0_) on the sheath near the constraint to ensure the shape passes through it during delivery. The configuration space is defined as ***c*** = [*s*_1_, *t*_1_, *r*_1_, *b*_1_, *t*_2_, *r*_2_, *b*_2_, *t*_3_, *r*_3_]^*T*^, where shaping, translational, rotational, and bending motions are abbreviated as *s, t, r*, and *b*.

To simplify clip pose modeling, each section can be built using the same model and combined using the standard Denavit–Hartenberg (DH) approach [29], which employs transformation matrices. The matrix ${\;}_{i-1}^{i}T$ represents the transformation from coordinate system *i* to *i* − 1, where, *i* = 1, 2, or 3. The transformation for a single section is shown in (1) where three DoFs are combined in series. Here, S* and C* represent the sine and cosine of angle (*), and *L*_*i*_ denotes the length of the straight region at the distal end. The 2D shape of each section is defined as (*f*_x_(*s*_*i*_, *b*_*i*_), *f*_z_(*s*_*i*_, *b*_*i*_)) in the bendable region or as (*f*_x_(*b*_*i*_, *b*_*i*_)+S*b*_*i*_*s*_*i*_, *f*_z_(*b*_*i*_, *b*_*i*_)+C*b*_*i*_*s*_*i*_) in the straight region, as previously described [27]. Here, *s*_*i*_ ranges from 0 to *b*_*i*_ for curvature along the bendable region and 0 to *L*_*i*_ for length along the straight region. The 2D position of the bendable tip, which is the junction between the bendable and straight regions, is represented as (*f*_x_(*b*_*i*_, *b*_*i*_), *f*_z_(*b*_*i*_, *b*_*i*_)), specified in the figure. The complete transformation model is obtained by multiplying the transformation matrices, as shown in (2). We simplified the 2D shape function of the GS to (*F*_*x*_, *F*_*z*_), which represents either the bendable or the straight region. The corresponding GS shape vector ***s*** is defined in (3). To convert the final transformation matrix into task-space coordinates, we define function *g*, which maps the 4×4 matrix to the task-space pose vector ***t*** = [*x, y, z, ϕ, θ, ψ*]^T^, representing position and Euler angles in 3D Euclidean space. The full kinematic model from ***c*** to ***t*** is thus $t=\text{g}\left({\;}_{0}^{3}T(c)\right)$.

(1) $$\begin{array}{l} {\;}_{i-1}^{i}T\left(t_{i},r_{i},b_{i}\right)\\ =\left[\begin{array}{cccc} \text{C}b_{i}\text{C}r_{i} & -\text{S}r_{i} & \text{S}b_{i}\text{C}r_{i} & f_{\text{x}}\left(b_{i},b_{i}\right)\text{C}r_{i}+L_{i}\text{S}b_{i}\text{C}r_{i}\\ \text{C}b_{i}\text{S}r_{i} & \text{C}rr_{i} & \text{S}bb_{i}\text{S}r_{i} & f_{\text{x}}\left(b_{i},b_{i}\right)\text{S}rr_{i}+L_{i}\text{S}b_{i}\text{S}r_{i}\\ -\text{S}b_{i} & 0 & \text{C}b_{i} & f_{\text{z}}\left(b_{i},b_{i}\right)+t_{i}+L_{i}\text{C}b_{i}\\ 0 & 0 & 0 & 1 \end{array}\right] \end{array}$$

(2) $${\;}_{0}^{3}T(c){=}_{0}^{1}T{\left(t_{1},r_{1},b_{1}\right)}_{1}^{2}T{\left(t_{2},r_{2},b_{2}\right)}_{2}^{3}T\left(t_{3},r_{3}\right)$$

(3) $$s=\begin{array}{ccc} {}[F_{x}\text{C}r_{1} & F_{x}\text{S}r_{1} & F_{z}+t_{1} \end{array}{]}^{\text{T}}$$

Next, we address the coupling between consecutive catheter handles. To control actuator positions corresponding to desired configurations, two mappings are defined: h : ℝ^9^ *→*ℝ^9^, ***c*** ↦ ***j*** (joint space, [0, *j*_1_, *j*_2_, …, *j*_7_, *j*_8_]^T^, representing catheter handle movements), and k : ℝ^9^*→*ℝ^9^, ***j*** ↦ ***m*** (actuator space, [0, *m*_1_, *m*_2_, …, *m*_7_, *m*_8_]^T^, representing actuator movements). In h, the DH modeling approach requires simultaneous translations of three catheter handles (joints *j*_1_, *j*_4_, and *j*_7_) to achieve translation *t*_1_ and coordinated rotations of three handles (*j*_2_, *j*_5_, *j*_8_) to achieve rotation *r*_1_. Similarly, *t*_2_ and *r*_2_ require compensatory motions from *j*_4_, *j*_7_ and *j*_5_, *j*_8_, respectively. The bending *b*_1_ and *b*_2_ are independently controlled by the knobs of the GS and DC, corresponding to joints *j*_3_ and *j*_6_. The functions *p*_GS_ and *p*_DC_ represent the linear relationship between knob rotation and the corresponding bending angles, accounting for dead zones and backlash (as detailed in prior research [27]). The calculation is shown in (4). In k, *n*_*i*_ denotes the transmission ratio for the *i*^*th*^ actuator, where *i* = 1…8. Actuators 1, 4, and 7 are integrated with the gear rack mechanism, while the remaining actuators are assembled with worm gear mechanisms. Actuators 2 and 3 are coordinated for GS rotation, whereas actuators 5 and 6 are coupled for DC rotation. The calculation is shown in (5). Hence, the complete kinematic model that computes the clip’s pose ***t*** depending on the actuator state ***m*** is given by $t=\text{g}({\;}_{0}^{3}T\left({\text{h}}^{-1}\left({\text{k}}^{-1}(m)\right)\right)$. Here, ∗^−1^ represents the inverse of function (∗).

**Fig. 4:**
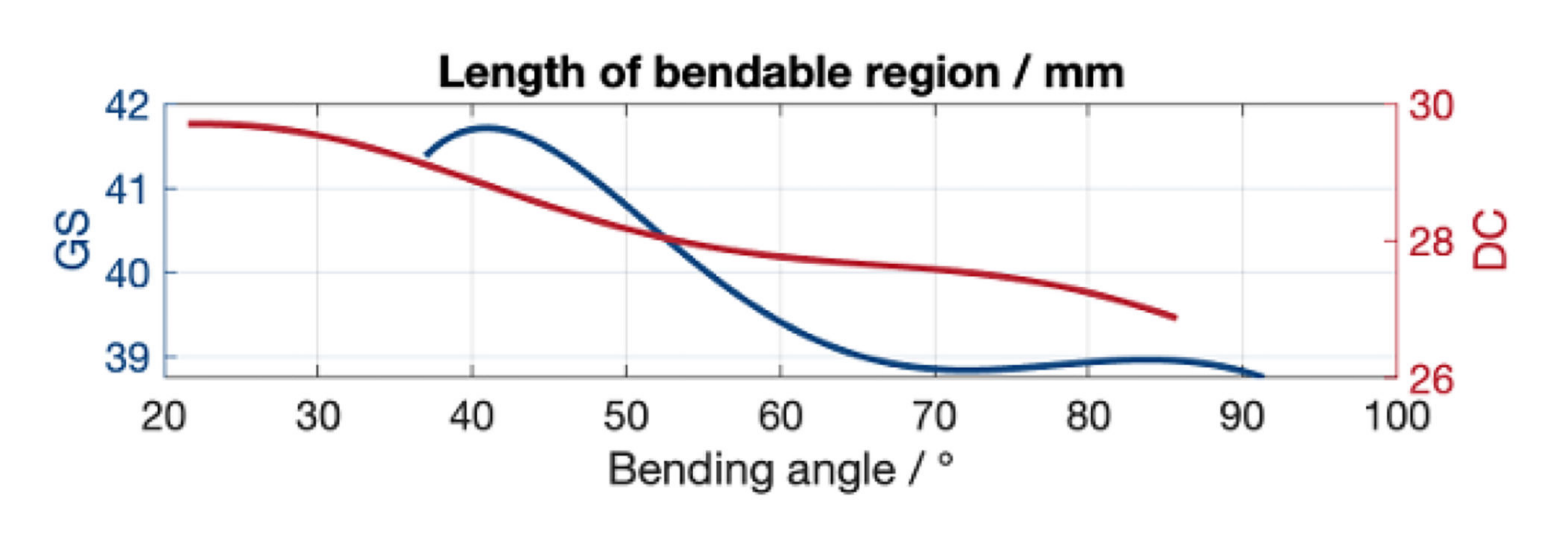
Lengths of the GS and DC bendable regions change nonlinearly during bending: from about 41.70 to 38.70 mm for the GS and from about 29.70 to 27.00 mm for the DC.

According to the study [27], the length of the bendable region changes during bending and must be compensated for in the model. The bendable region length of the GS is calculated as the sum of the lengths of two curved segments. Each segment length is determined by the product of the curvature angle in radians, *θ*_GS_(*b*_1_) or *b*_1_ − *θ*_GS_(*b*_1_), and the corresponding curvature radius *r*_GS1_(*b*_1_) or *r*_GS2_(*b*_1_). The total length is given by: *l*_GS_(*b*_1_) = *r*_GS1_(*b*_1_)*θ*_GS_(*b*_1_) + *r*_GS2_(*b*_1_)(*b*_1_ − *θ*_GS_(*b*_1_)). The calculation for DC follows the same approach. As shown in Fig. 4, the GS length decreases from approximately 41.70 to 38.70 mm, and the DC length reduces from about 29.00 to 27.00 mm as the bending angle increases. We then define the compensation as *q*_GS_(*b*_1_) = (*l*_GS_(*b*_1_) − *L*_GS_(*b*_1_)), where *L*_GS_(*b*_1_) is the length without bending. A similar expression is used for the DC as *q*_DC_(*b*_2_). The function h(***c***) is updated to h^*′*^(***c***) in (6).

(4) $$\begin{array}{l} j=\text{h}(c)=[\begin{array}{c} 0\;t_{1}\;r_{1}\;p_{\text{GS}}\left(b_{1}\right)\;t_{1}+t_{2}\;r_{1}+r_{2} \end{array}\\ {\;p_{\text{DC}}\left(b_{2}\right)\;t_{1}+t_{2}+t_{3}\;r_{1}+r_{2}+r_{3}]}^{\text{T}} \end{array}$$

(5) $$\begin{array}{l} m=[\text{k}(j)=\begin{array}{ccc} 0 & n_{1}j_{1} & n_{2}j_{2} \end{array}\begin{array}{cc} n_{3}\left(j_{2}+j_{3}\right) & n_{4}j_{4} \end{array}\\ \;\begin{array}{cccc} n_{5}j_{5} & n_{6}\left(j_{5}+j_{6}\right) & n_{7}j_{7} & n_{8}j_{8} \end{array}{]}^{\text{T}} \end{array}$$

(6) $$\begin{array}{l} j={\text{h}}^{\prime }(c)=[\begin{array}{cccc} 0 & t_{1} & r_{1} & p_{\text{GS}}\left(b_{1}\right) \end{array}\\ \;t_{1}+t_{2}+q_{\text{GS}}\left(b_{1}\right)\;r_{1}+r_{2}\;p_{\text{DC}}\left(b_{2}\right)\\ \;{t_{1}+t_{2}+t_{3}+q_{\text{GS}}\left(b_{1}\right)+q_{\text{DC}}\left(b_{2}\right)\;r_{1}+r_{2}+r_{3}]}^{\text{T}} \end{array}$$

### C Image-Space Pose Control Strategy

**Fig. 5:**
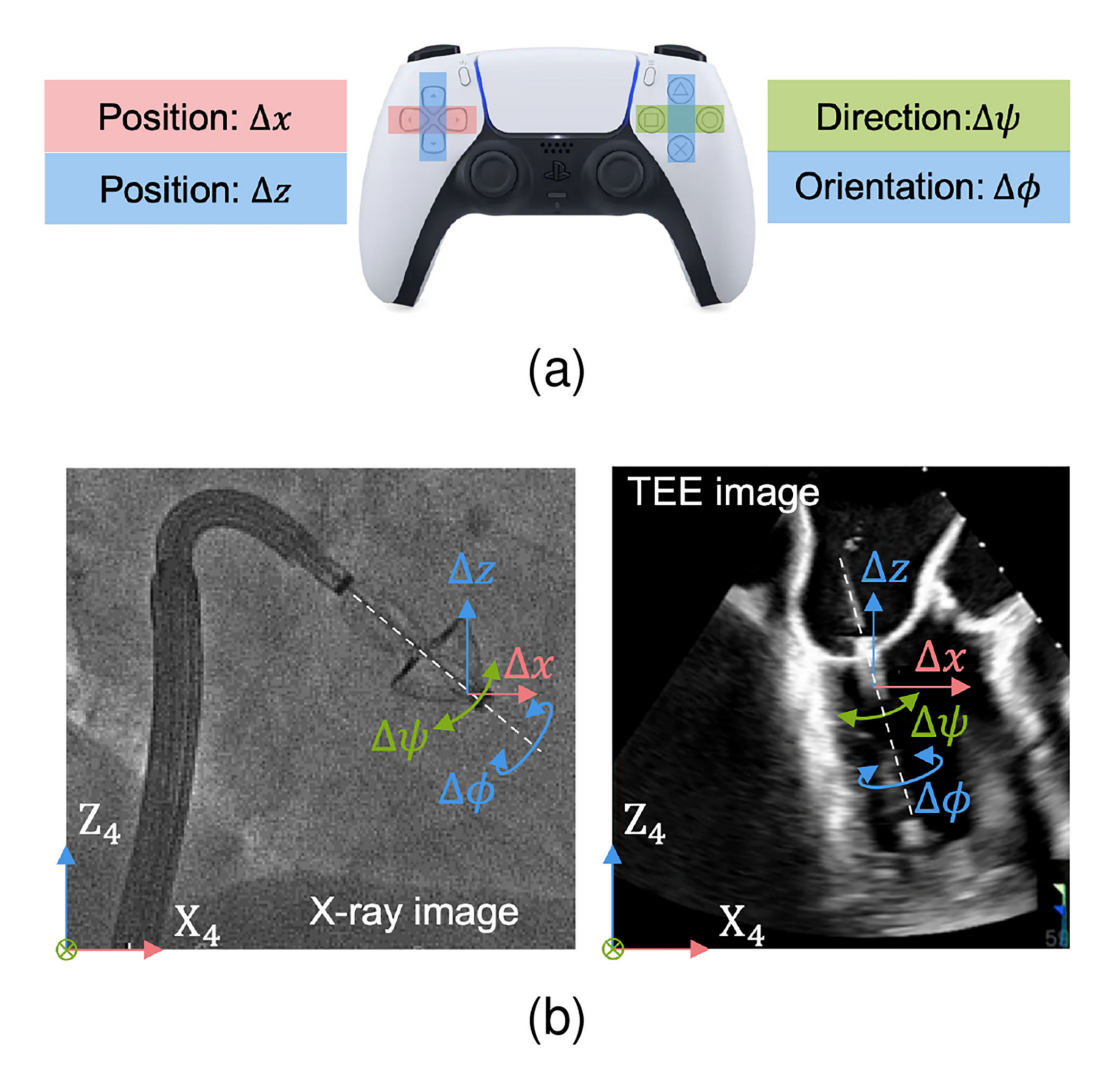
Independent control strategy based on registered images (a) Command assignments on the PS5 controller. (b) Clip response in registered image coordinates: X-ray (left, from [30], CC-BY) and ultrasound (right, from [31], CC-BY).

To adjust the clip’s position, direction, and orientation independently and intuitively within 2D image coordinates, we use a PlayStation 5 Dualsense wireless controller (PS5, Sony, Minato, Tokyo, Japan) to send commands separately. Figure 5a illustrates the command assignments on the PS5: the left joystick controls position adjustments Δ*x* and Δ*z*, while the right joystick controls direction Δ*ψ* and orientation Δ*ϕ*. The corresponding clip movements within the image coordinates *{*X_4_Y_4_Z_4_*}* are shown in Fig. 5b. Specifically, Δ*x* and Δ*z* translate along X_4_ and Z_4_ axes. Δ*ψ* rotates about an axis passing through the clip tip and is parallel to the Y_4_ axis, while Δ*ϕ* rotates about the Z_3_ axis of clip coordinates. Adjustment axes are color-coded: X (red), Y (green), and Z (blue).

To apply displacements from the PS5 controller, a 3×3 rotation matrix, ${\;}_{\text{base}}^{\text{image}}R$, is first computed to transform image coordinates (X-ray or TEE) to the robot base frame. For X-ray imaging, this matrix is parameterized by the LAO-RAO and CRA-CAU angles of the X-ray C-arm as in previous work [26]. For TEE images, the approach from [32] registers TEE with X-ray fluoroscopy via 3D probe reconstruction and 2D-3D image registration. The TEE-to-robot base transformation is then obtained by combining this registration with the X-ray-to-robot base transformation. As the main focus of this work is on design, modeling, pose control, and validation, the details of the registration process are not given here.

The clip’s position displacement in image coordinates, ${\;}_{\text{image}}^{\text{clip}}P(\text{Δ}x,\text{Δ}z)={\left[\text{Δ}x,0,\text{Δ}z\right]}^{T}$, is updated based on translational movements. Its two rotational movements are represented by ***R***_y_(Δ*ψ*) and ***R***_z_(Δ*ϕ*), as shown in (7) and (8.). Initially, the expected position and rotation matrix are set to the current values: ${\;}_{\text{base}}^{\text{clip}}P_{\text{exp}}^{0}={\;}_{\text{base}}^{\text{clip}}P_{\text{cur}}\;\text{and}{\;}_{\text{base}}^{\text{clip}}R_{\text{exp}}^{0}={\;}_{\text{base}}^{\text{clip}}R_{\text{cur}}$ As the system transitions from state *i* to state *i* + 1, updates are driven by the PS5 controller, as defined in (9) and (10). The task space pose vector ***t***_exp_ can be calculated using the function g. The reference point (*x*_0_, *y*_0_, *z*_0_) on the GS was defined manually using (3), and the expected shape ***s***_exp_ is designed to pass through the reference point, as given in (11).

(7) $$R_{\text{y}}(\text{Δ}\psi )=\left[\begin{array}{ccc} \text{CΔ}\psi & 0 & \text{SΔ}\psi \\ 0 & 1 & 0\\ -\text{SΔ}\psi & 0 & \text{CΔ}\psi \end{array}\right]$$

(8) $$R_{\text{z}}(\text{Δ}\phi )=\left[\begin{array}{ccc} \text{CΔ}\phi & -\text{SΔ}\phi & 0\\ \text{SΔ}\phi & \text{CΔ}\phi & 0\\ 0 & 0 & 1 \end{array}\right]$$

(9) $${\;}_{\text{base}}^{\text{clip}}P_{\text{exp}}^{i+1}{=}_{\text{base}}^{\text{clip}}P_{\text{exp}}^{i}{+}_{\text{base}}^{\text{image}}R_{\text{image}}^{\text{clip}}P(\text{Δ}x,\text{Δ}z)$$

(10) $${\;}_{\text{base}}^{\text{clip}}R_{\text{exp}\;}^{i+1}{=}_{\text{base}}^{\text{image}}R\left(R_{\text{y}}{\left(\text{Δ}\psi \right)}_{\text{image}}^{\text{clip}}R_{\text{exp}}^{i}R_{\text{z}}(\text{Δ}\phi )\right)$$

(11) $$\{\begin{array}{c} t_{\exp}=\text{g}(\left[\begin{array}{cc} {\;}_{\text{base}}^{\text{clip}}R_{\exp} & {\;}_{\text{base}}^{\text{clip}}P_{\exp}\\ 0_{1\times 3} & 1 \end{array}]\right)\\ s_{\exp}={\left[x_{0},y_{0},z_{0}\right]}^{\text{T}} \end{array}$$

### D Closed-Loop Controller Design

**Fig. 6:**
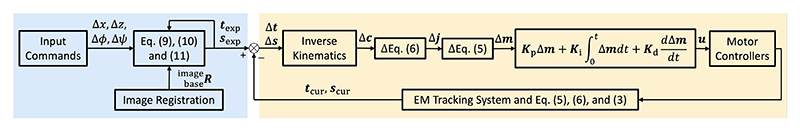
Control framework overview: The blue panel illustrates an image-space pose control strategy, and the yellow panel demonstrates the PID-based closed-loop control.

A closed-loop controller is implemented to address errors from modeling inaccuracies. It focuses on clip positioning accuracy, as GS shape control has already been studied in [26]. The control framework is illustrated in Fig. 6. The blue box represents the image-space pose control commands described in Section II-C. The expected pose (***t***_exp_) is compared with the current pose (***t***_cur_) which is acquired from an electromagnetic (EM) tracker (Aurora, Northern Digital Inc., Waterloo, Ontario, Canada), to get the pose error (Δ***t***). Similarly, the expected shape (***s***_exp_) is compared with the current shape, derived using (3), to determine the shape error (Δ***s***). To convert the task space variables ***t*** and ***s*** to the configuration space ***c***, we apply differential kinematics. This establishes the relationship between the clip pose, shape, and the corresponding configurations based on the Jacobian matrix ($J={\left[J_{\text{t}}^{\text{T}},J_{\text{s}}^{\text{T}}\right]}^{\text{T}}$, where ***J***_t_ = *∂t/∂****c*** and ***J***_s_ = *∂**s**/∂****c***). Then the configuration difference Δ***c*** is approximately calculated using damped least squares to improve robustness.

(12) $$\text{Δ}c=J^{\text{T}}{\left(JJ^{\text{T}}+\lambda^{2}I_{9\times 9}\right)}^{-1}\left[\begin{array}{l} \text{Δ}t\\ \text{Δ}s \end{array}\right]$$

where *λ* and ***I***_9×9_ are the damping factor and identity matrix.

The difference in actuator space is calculated by Δ(6) and Δ(5), where Δ* represents the deviation form of the function *. Then we apply the PID control method. The control law, ***u***, is designed as: $u=K_{\text{p}}\text{Δ}m+K_{\text{i}}\int_{0}^{t}\text{Δ}mdt+K_{\text{d}}\frac{d\text{Δ}m}{dt}$, where ***K***_p_, ***K***_d_, and ***K***_i_ are gains (9×9 nonnegative diagonal matrices). ***u*** is then tracked by low-level motor controllers at each actuator. This control strategy continuously adjusts ***u*** to minimize tracking errors and to follow commands over time.

### E Simulation and Experiment Setup

We setup simulations and experiments to validate the control framework step by step. The simulations evaluate the effectiveness of independent pose control in image space. This is followed by an experimental comparison of control accuracy between the proposed and open-loop control methods.

#### 1) Image-Space Pose Control Simulations

**Fig. 7:**
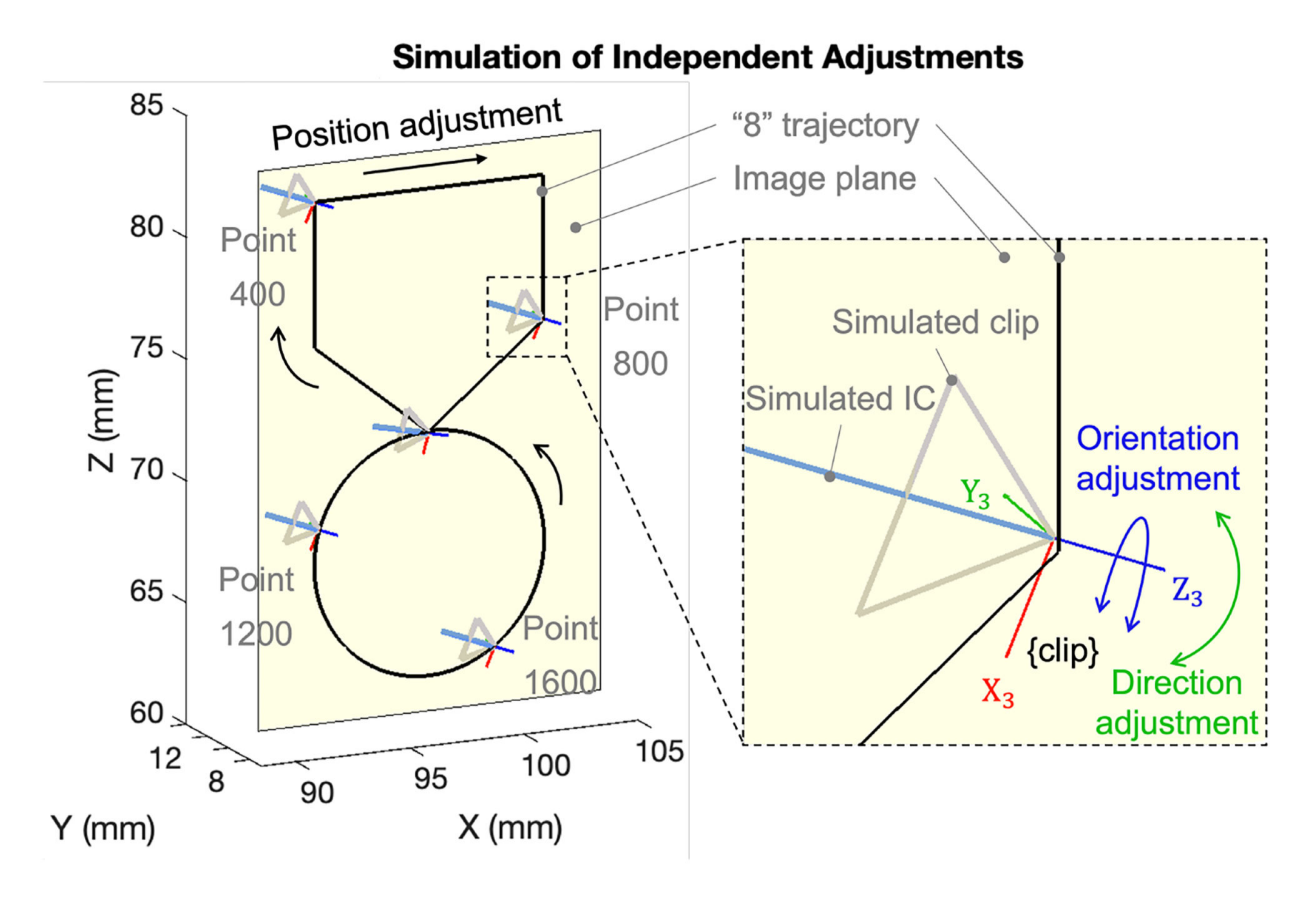
Demonstration on the imaging plane (RAO 0°, CAU 0°) showing independent adjustments of position (black), direction (green), and orientation (blue).

Simulations were developed using C++ to validate the independent control functionality across various image views. Figure 7 shows one simulation on the imaging plane (RAO 0°, CAU 0°), displayed in the robot base coordinate system {X_0_Y_0_Z_0_}. A predefined 20×10 mm^2^ figure-eight (“8”) trajectory was used, consisting of five straight paths on the top and a curved path on the bottom. Each straight path was discretized into 200 evenly spaced points, and the curved path into 1000 points. The distance between neighboring points ranges from about 0.025 to 0.050 mm. The robot was commanded to follow the trajectory at a fixed interval of 60 ms per point. At points 0, 400, 800, 1200, and 1600, we perform direction and orientation adjustments: the direction is modified incrementally from 0° to 10° and then returned to 0°, followed by a similar orientation change from 0° to 15° and back. This approach ensured that only one adjustment was applied at a time, allowing for easy verification of the independent control performance. The sequence was completed in 240 seconds. During movement, the simulations stopped if the Euclidean norm of Δ***s*** was greater than 0.10 mm. To broaden the evaluation, three sequences were performed using distinct X-ray image views: (RAO 0°, CRA 0°), (RAO 20°, CRA 20°), and (LAO 40°, CAU 10°). Performance metrics include:

- Distance: Euclidean distance from the clip’s current location to the simulation start point.
- Direction: Projection of the clip’s Z-axis onto the imaging plane.
- Orientation: Rotation angle of the clip around its Z-axis.

Ideal independent control was indicated when only the adjusted parameter changed while the other two remained stable. Performance was evaluated by recording the maximum deviations in the other two non-adjusted parameters. For instance, during direction adjustments, the maximum changes in distance and orientation were measured.

#### 2) Clip Positioning Experiments

**Fig. 8:**
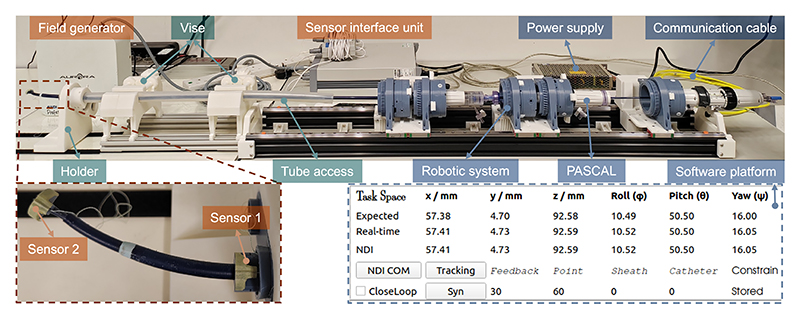
Experimental setup for clip positioning. It includes the robotic system (blue) with a monitoring user interface (UI), a support system (dark teal) simulating trans-femoral access, and a measurement system (orange) utilizing two NDI 6-DoF EM sensors to calculate the clip’s pose. The NDI system is integrated into the UI, enabling simultaneous data acquisition.

The experimental platform (Fig. 8) was developed to evaluate clip positioning accuracy and consists of three main components: the robotic system (blue), the support system (dark teal), and the measurement system (orange). The robotic system was designed and assembled as described in Section II-A, with the PASCAL delivery system pre-installed. It is connected to the controller via a CAN bus communication cable. A custom software platform facilitates real-time monitoring of actuator parameters (position, speed, and limits) and provides visualization of the expected and actual GS shape (***s***_exp_ and ***s***_cur_), as well as the clip pose (***t***_exp_ and ***t***_cur_). To simulate trans-femoral access, the support platform includes a tube, two vises, and a holder structure for insertion. For measurement, the system employs two 6-DoF EM sensors, a sensor interface unit, and a field generator to track the clip pose. Sensor 1, mounted on the holder structure, represents the base coordinates {X_0_Y_0_Z_0_}, while Sensor 2, attached to the clip’s end, represents the clip coordinates {X_3_Y_3_Z_3_}. The clip pose is calculated by determining the transformation matrix from Sensor 2 to 1. The NDI system is integrated into the software, and the data is processed using an average filter to enhance signal stability. This integration allows synchronized collection of expected data from the robot and actual data from the NDI at 40 Hz.

We observed that orientation adjustment is inherently independent, as it is achieved solely by rotating the IC (*r*_3_). Therefore, our experiments focused on evaluating the position and direction adjustments to assess the control accuracy of the closed-loop controller compared to open-loop control. For position adjustments, we used the same predefined path as in the simulations, which was generated and loaded into the software platform. For direction adjustments, the orientation was sequentially adjusted by increasing the angle by 10°, then decreasing it to -10°, and finally returning to 0°. Control accuracy was assessed by measuring the distance between the actual pose, tracked by the NDI sensors, and the target pose. Angular error was calculated as the scalar part of the relative quaternion, indicating the minimum rotational difference between actual and expected 3D orientations.

## III Results and Discussion

### A Image-Space Pose Control Simulations

Based on measurements from real catheters, straight region lengths are defined as *L*_1_ = 11.5 mm, *L*_2_ = 5 mm, and *L*_3_ = 0 mm. A summary of maximum deviations is provided in Table II. The results indicate that orientation adjustment is the most independent, causing minimal changes in distance (0.09 mm) and direction (0.05°) in all imaging views. This independence is attributed to the fact that the orientation adjustment is achieved only by modifying the rotation of the IC (*r*_3_). Position adjustments introduce minor changes in direction and orientation, approximately 0.15° and 0.15°, which are likely due to computational errors in the control loop. However, direction adjustments significantly affect orientation, with changes ranging from 0.20° to 2.69°. This effect is mainly because the projection of the clip’s Z-axis on the imaging plane inherently influences the clip’s rotation. To mitigate this issue, it is recommended to perform direction adjustments before orientation adjustments. This sequence minimizes coupling effects and improves overall control accuracy.

### B Clip Positioning Experiments

After systematic tuning, the parameters in (12) are set as follows: *λ* = 0.3, ***K***_p_ = diag(0, 0.2, 0.3, 0.35, 0.2, 0.3, 0.35, 0.2, 0.3), ***K***_i_ = diag(0, 0.001,…, 0.001), and ***K***_d_ = **0**_9×9_.

**Fig. 9:**
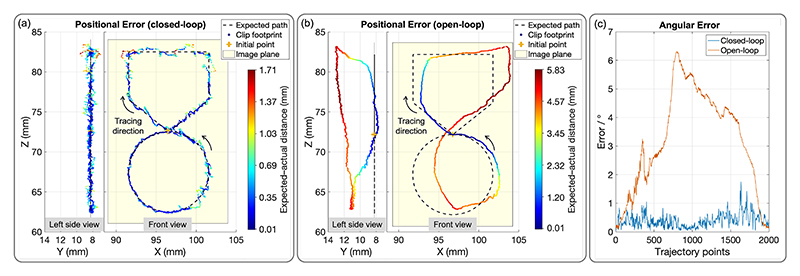
Position adjustment: Positional and angular error comparison between closed-loop and open-loop control.

The control period is 60 ms. The transmission ratios are described in II-A. The results of position adjustments under closed-loop and open-loop control are presented in Fig. 9. Specifically, Fig. 9a depicts the position errors in closed-loop control, while Fig. 9b illustrates the position errors in open-loop control. The dashed line represents the expected path, the colored markers indicate the clip’s actual path, with colors transitioning from blue to red as the distance increases. The yellow plane represents the image view. Additionally, Fig. 9c compares the angular errors between the two control methods.

In open-loop control, the positional errors are non-uniform and vary along the trajectory. In the front view (X-Z plane), the error gradually increases from the initial point, with a slight drift in the positive X direction. The drift peaks at the top right corner, where a drift in the positive Z direction also occurs. After the trajectory transitions from the horizontal path to the right vertical path, the error reaches its maximum (Max: 5.83 mm) due to these accumulated drifts. As the path returns toward the initial point, the error gradually decreases. After passing the initial point again, a positive X-direction offset is observed on the left circle path. The error then continues to decrease as the trajectory completes. Overall, errors along the Z-axis are minimal, while the trajectory exhibits a slight positive X-direction drift. Additionally, the upper horizontal path is inclined, primarily due to misalignment between the inner diameter of the DC and the outer diameter of the IC, causing the IC to exit the DC slightly higher than the ideal alignment. In the left side view (Y-Z plane), the error in the positive Y direction increases and then decreases, with noticeable deviations near the transitions from the horizontal path to the right vertical path and from the left circle to the right circle path. These deviations are attributed to two factors: (1) catheter tip twisting during bending, as observed in our previous study [27], which was simplified out in the current model for control efficiency; and (2) slight torsional deformation of the rubber material when excessive knob turning occurred. This twisting caused the clip to shift along the Y-axis. The RMSE and standard deviation (SD) in open-loop control are 3.95 and 1.88 mm, respectively. In closed-loop control, these values are significantly reduced to 0.44 and 0.26 mm, demonstrating improved accuracy and consistency. However, large errors (Max: 1.71 mm) are still observed in the lower right circle path and during transitions from the left vertical path to the horizontal path, as well as from the horizontal path to the right vertical path. These regions of high error coincide with similar locations in open-loop control, primarily due to vibrations along the long shaft of the catheters. The longer the shaft, the greater the susceptibility to disturbances, which amplifies the errors in these areas. The angular error analysis in open-loop and closed-loop control aligns well with the path-based observations. In open-loop control, the angular error increases steadily along the trajectory, peaking at 6.32° before gradually decreasing. This aligns with the observed trajectory deviations in the X-Z plane, particularly during the transitions. The partial reduction in error toward the end suggests a slight correction as the system nears the initial point. The RMSE and SD for open-loop control are 3.66° and 1.75°, respectively. In contrast, the angular error in closed-loop control remains consistently low, typically below 1°, with minor fluctuations. Peaks (Max: 1.76°) occur primarily during challenging transitions, but these deviations are significantly smaller than those in open-loop control. The RMSE and SD are reduced to 0.46° and 0.23°, demonstrating the effectiveness of feedback in maintaining angular alignment and mitigating twisting and material deformation effects. All results are summarized in Table III.

**Fig. 10:**
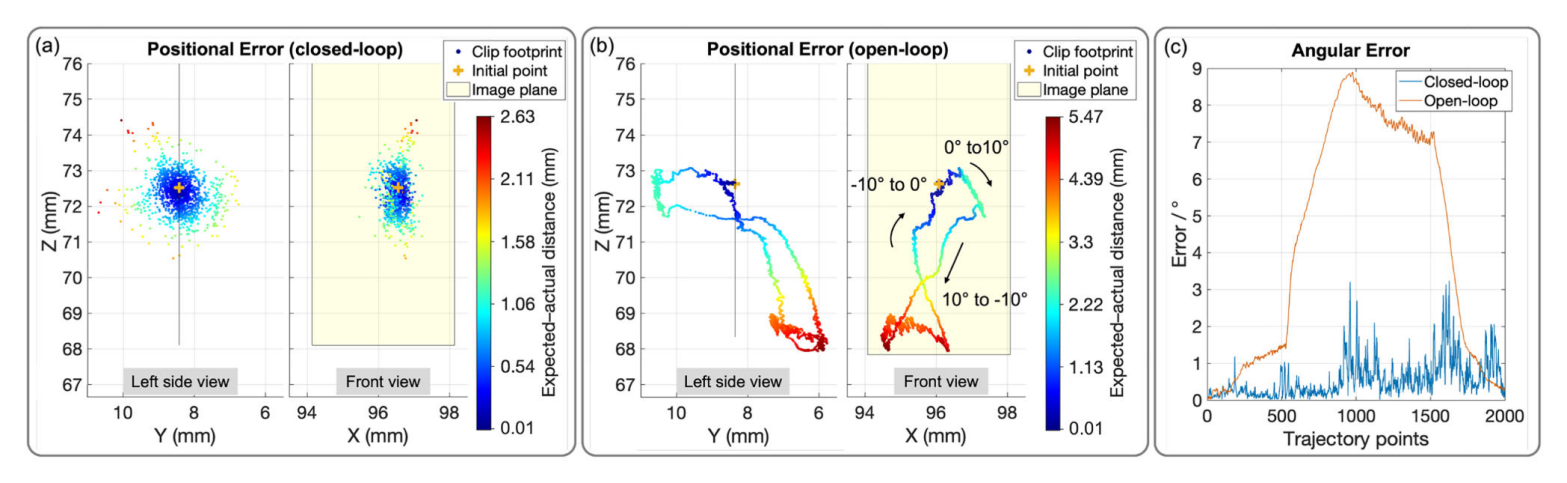
Direction adjustment: Positional and angular error comparison between closed-loop and open-loop control.

Figure 10 presents the positional and angular errors during direction adjustments. The angular error increases steadily in Phase 1 (from 0° to 10°) and grows dramatically in the first half of Phase 2 (from 10° to -10°), peaking at 8.90°. This significant increase is likely attributed to misalignment and hysteresis effects within the catheters during reversal. In the second half of Phase 2, the error decreases slightly as the system stabilizes. During the final reversal in Phase 3 (from -10° to 0°), the error decreases rapidly as the system returns to the neutral direction. This reduction is passive and not intentional, resulting from the direction coming back closer to the expected direction. The RMSE and SD of the angular error are 5.35° and 3.19°, respectively. Closed-loop control demonstrates smaller angular errors (< 2° most of the time) but exhibits some oscillations and spikes, especially during direction transitions in the second-half adjustments. These spikes are likely due to dynamic effects or delays in feedback correction. In comparison, open-loop control results in unbounded error growth, emphasizing the effectiveness of closed-loop control in stabilizing performance after disturbances. Ideally, direction adjustments should not induce positional changes. However, the experimental results indicate unintended clip movement. For clarity, the footprint trajectory is annotated with the corresponding phases of direction adjustment. Similarly to angular errors, the positional error increases steadily in Phase 1, rises sharply in the first half of Phase 2 (Max: 5.47 mm), stabilizes in the second half of Phase 2, and decreases in Phase 3 as the system returns closer to the initial point. Sudden changes occur during the transitions between phases. The RMSE and SD of the positional error are 3.42 mm and 1.63 mm, respectively. Under closed-loop control, these are significantly reduced (Max: 2.63 mm, RMSE: 0.59 mm, SD: 0.39 mm). Notably, the angular errors in open-loop and closed-loop control during position adjustments are lower than those observed in direction adjustments. This could be due to direction adjustments causing more rubber deformation.

Open-loop control exhibited large, unbounded errors in both position and direction adjustments, particularly during reversals. These errors were due to factors such as misalignment between catheters, the limitations of a simplified model, and torsional deformation of the rubber material. In contrast, closed-loop control effectively reduced both positional and angular deviations. Given that the anatomical suitability is defined by a coaptation length greater than 2 mm and depth less than 11 mm [33], the positioning errors reported here are considered clinically acceptable. In real procedures, success is determined by effective leaflet capture and MR reduction, which are assessed using real-time TEE and color Doppler imaging [4]. If leaflet grasp or positioning is inadequate, the clip can be unlocked, repositioned, and reattempted until the interventional team is satisfied [34]. Further optimization, such as the design of smoother transition paths, may help reduce maximum positioning errors, thereby improving procedural efficiency and minimizing the need for repositioning.

## IV Conclusion and Future Work

In this paper, we present a compact 8-DoF robot for PASCAL, a TDC catheter system designed for M-TEER. The robot enables independent and precise control of the clip’s position, direction, and orientation in image space. This is achieved through a novel control framework that combines a pose mapping strategy relative to image views with a Jacobian-based PID controller for closed-loop tracking. The simulation results demonstrated effective independent control, with only direction adjustments showing minor coupling with orientation (maximum deviation: 2.69°). This can be minimized by adjusting the direction first in real cases. Positioning experiments showed that closed-loop control effectively mitigates catheter misalignment and rubber deformation, achieving average positional and angular RMSEs of 0.52 mm and 0.61°. These values are considered clinically acceptable. This work focuses on technical feasibility, though some challenges remain for clinical translation. First, the feedback relies on an NDI system, which may be susceptible to interference in the catheterization laboratory. Second, experiments were performed with the clip moving in free space; contact with a phantom or cardiac tissue may influence control performance. Despite these limitations, this work advances the state-of-the-art in image-space control of TDC catheters.

In future work, we aim to explore feedback strategies based on imaging modalities, enabling real-time clip control directly from images. To address the larger errors observed during path transitions, we will also generate a smooth path for MV navigation. Finally, we will evaluate the robot’s performance in realistic and diverse anatomical scenarios to ensure its reliability and adaptability in clinical applications.

## Figures and Tables

**Table I:** State-of-the-Art Tendon-Driven Concentric Robots for MV Interventions and Other Purposes (2020–2025)

Reference	Modeling	DoFs	Target	Controller (errors)	Image	Application
[12], Zhang, 2022	CR	3	Tip position	PID (1.29 mm)	No	Mitral repair
[13], Bicchi, 2025	CC	3	Tip position	P (1.92 mm)	No	Mitral repair
[14], Qi, 2023	CC	4	Tip position, orientation	PID (1.00 mm, 2.00°)	No	Mitral implant
[15], Nayar, 2024	CC	4	Joints	PID	Yes	Mitral implant
[16], SurgiPulse, 2024	—	—	Joints	—	Yes	Mitral repair
**This robotic system**	PCC + PJ	8	Tip pose	PID (0.52 mm, 0.61°)	Yes	Mitral repair
[17], Jeong, 2020	CC (adjustable)	4	Tip position	— (9.60 mm)	No	Endovascular
[18], Sarma, 2022	CC (adjustable)	5	Tip position	PID (5.34 mm)	No	Endovascular
[19], Chitalia, 2023	CR	4	Tip position	— (5.55 mm)	No	Endoscopic/Intracardiac
[20], Yamamoto, 2025	CR	6	Tip position	— (< 5 mm)	No	Endoscopic endonasal
[21], Wei, 2023	CC	5	Tip position	— (1.50 mm)	No	Minimally invasive
[22], Zhang, 2025	Optimized PCC	7	Tip position	— (1.78 mm)	No	Minimally invasive
[23], Wang, 2024	CC	6	Tip pose	P (< 2 mm)	No	Cardiac ablation
[24], Ma, 2023	CC	9	Tip pose (shape)	— (1.60 mm, 4.50°)	No	Inspection/Minimally invasive
[25], Gao, 2024	CC	6	Tip pose (shape)	— (2.65 mm)	No	Oropharyngeal swab sampling

*Acronyms*: CR, Cosserat rod; CC, constant curvature; PCC, piecewise constant curvature; PJ, pseudo joints. *Explanation*: Errors are given as RMSE or as the mean L1/L2 norm, averaged over all experiments when applicable.

**Table II:** Maximum Deviations of Non-Targeted Movements During a Single Adjustment Across Simulated X-Ray Views

Maximum deviations	Position adjustment		Direction adjustment		Orientation adjustment	
	direction / °	orientation / °	distance / mm	orientation / °	distance / mm	direction / °
RAO 0°, CAU 0°	0.10	0.03	0.43	0.20	0.02	0.01
RAO 20°, CRA 20°	0.15	0.07	0.01	1.23	0.09	0
LAO 40°, CAU 10°	0.15	0.15	0.10	2.69	0.02	0.05

**Table III:** Positional and Angular Errors of Clip Positioning

Errors	Positional error / mm			Angular error / °		
	Max	RMSE	SD	Max	RMSE	SD
Position (open)	5.83	3.95	1.88	6.32	3.66	1.75
Position (closed)	1.71	0.44	0.26	1.76	0.46	0.23
Direction (open)	5.47	3.42	1.63	8.90	5.35	3.19
Direction (closed)	2.63	0.59	0.39	3.24	0.75	0.51
